# COVID-19 vaccine acceptance and hesitancy among primary healthcare workers in Singapore

**DOI:** 10.1186/s12875-022-01693-z

**Published:** 2022-04-15

**Authors:** Sky Wei Chee Koh, Yiyang Liow, Victor Weng Keong Loh, Seaw Jia Liew, Yiong-Huak Chan, Doris Young

**Affiliations:** 1 National University Polyclinics, National University Health System, Singapore, Singapore; 2 Department of Family Medicine, Yong Loo Lin School of Medicine, National University of Singapore, Singapore, Singapore; 3 Biostatistics Unit, Yong Loo Lin School of Medicine, National University of Singapore, Singapore, Singapore

**Keywords:** COVID-19 vaccine, Vaccine hesitancy, Healthcare workers, Primary care, General practice, Singapore

## Abstract

**Background:**

Factors affecting COVID-19 vaccine acceptance and hesitancy among primary healthcare workers (HCW) remain poorly understood. This study aims to identify factors associated with vaccine acceptance and hesitancy among HCW.

**Methods:**

A multi-centre online cross-sectional survey was performed across 6 primary care clinics from May to June 2021, after completion of staff vaccination exercise. Demographics, profession, years working in healthcare, residential status, presence of chronic medical conditions, self-perceived risk of acquiring COVID-19 and previous influenza vaccination were collected. HCW who accepted vaccine were then asked to rank their top 5 reasons for vaccine acceptance; HCW who were vaccine hesitant had to complete the 15-item 5C scale on psychological antecedents of vaccination.

**Results:**

Five hundred fifty seven out of 1182 eligible HCW responded (47.1%). Twenty nine were excluded due to contraindications. Among 528 respondents, vaccine acceptance rate was 94.9% (*n* = 501). There were no statistically significant differences in COVID-19 vaccine acceptance between sex, age, ethnicity, profession, number of years in healthcare, living alone, presence of chronic diseases, self-perceived risk or previous influenza vaccination. The top 3 reasons for COVID-19 vaccine acceptance ranked by 501 HCW were to protect their family and friends, protect themselves from COVID-19 and due to high risk of acquiring COVID-19 because of their jobs. HCW with suspected or confirmed COVID-19 exposure were 3.4 times more likely to rank ‘high risk at work’ as one of the top reasons for vaccine acceptance (χ^2^ = 41.9, *p* < 0.001, OR = 3.38, 95%C.I. 2.32–4.93). High mean scores of ‘Calculation’ (5.79) and low scores for ‘Constraint’ (2.85) for 5C components among vaccine hesitant HCW (*n* = 27) highlighted that accessibility was not a concern; HCW took time to weigh vaccine benefits and consequences.

**Conclusion:**

COVID-19 vaccine hesitancy is a minute issue among Singapore primary HCW, having achieved close to 95% acceptance rate. COVID-19 exposure risk influences vaccine acceptance; time is required for HCW to weigh benefits against the risks. Future studies can focus on settings with higher hesitancy rates, and acceptance of booster vaccinations with the emergence of delta and omicron variants.

**Supplementary Information:**

The online version contains supplementary material available at 10.1186/s12875-022-01693-z.

## Background

As of March 2022, more than 475 million individuals worldwide have been infected with Coronavirus Disease 2019 (COVID-19) with about 6.1 million deaths since its emergence in late 2019 [1]. Vaccination is a key public health strategy because it has been shown to be effective in reducing risk of infection and severe disease [2, 3]. Healthcare workers (HCW) are defined as paid or unpaid persons engaged in actions whose primary intent is to enhance health [4]. They are at increased risk of exposure due to the nature of their work, and therefore achieving high vaccination rates in this group is critical. They are also the most trusted advisors; improving knowledge and confidence in vaccines have been shown to increase willingness to recommend vaccines and influence their patient’s decisions [5]. Therefore, understanding vaccine acceptance and hesitancy amongst HCW is necessary.

Vaccine hesitancy is defined by the World Health Organisation (WHO) as the delay in acceptance or refusal of vaccination despite availability [6]. At its core, vaccine hesitancy results from the decision-making process after considering a multitude of factors which influence that decision. Frameworks such as the 3Cs model, Working Group Determinants of Vaccine Hesitancy Matrix and 5Cs psychological antecedents have been theorised in an attempt to classify these factors contributing to vaccine hesitancy [6, 7]. With the introduction of COVID-19 vaccines, vaccine acceptance rates among HCW was found to range from 27.7 to 78.1% in a few systematic reviews, with main concerns regarding safety or potential side effects [8, 9]. While some studies noted that age, sex, occupation were positive predictors for hesitancy, some found that previous influenza vaccination and self-perceived risk were facilitators for vaccine acceptance [10–13]. In Asia, a study have shown that the majority of HCW are willing to receive the COVID-19 vaccination, with perceived susceptibility, low potential risk of vaccine harm and pro-socialness as main drivers [14].

An island city-state with a population of more than 5.7 million, Singapore encountered its first case of COVID-19 in January 2020. At the forefront of the local response to COVID-19, primary care forms the foundation of the Singapore healthcare system, provided by over 1700 private general practitioner clinics and 20 government subsidised primary care clinics (polyclinics) from 3 healthcare clusters [15]. These 20 public primary care clinics located islandwide account for 21% of all primary care outpatient visits in the country [15]. Primary care providers were involved in setup and operations of community isolation facilities, public health preparedness clinics (testing, management and surveillance for COVID-19 cases) and act as sites for COVID-19 vaccination [16]. Singapore first started HCW vaccination against COVID-19 with the Pfizer-BioNTech/Comirnaty vaccine in end December 2020. With community control efforts helmed by an efficient and united COVID-19 multi-ministry task force coupled with good communication and a strong primary care, Singapore was able to keep its death rate low with a high vaccination rate of 92% in March 2022 [17]. To examine the potential lessons learnt in this journey, it will be vital to study the perspective of primary care HCW who serve on the frontlines during the initial vaccine rollout; having a greater understanding of HCW thoughts and behaviours will definitely shed light on patients’ vaccine decisions and influence policies. In order to do this, a questionnaire-based study implemented just after conclusion of staff vaccination exercise will be more accurate in ascertaining hesitancy after actual uptake, compared to other studies performed prior to vaccine rollout (which measures intention to vaccinate). Therefore, this study seeks to understand COVID-19 vaccine acceptance and hesitancy among HCW in a primary healthcare cluster in Singapore by adopting the abovementioned methodology to elevate the strength of results, bringing it closer to the true definition of vaccine hesitancy.

## Method

### Study population and setting

The study involved HCW from 6 publicly funded polyclinics who are part of a healthcare cluster in the western region of Singapore. They provide treatment for acute illnesses, management of chronic diseases, women and children health services such as immunizations and dental care. Each clinic comes with its own laboratory, radiological and pharmacy services. A cross-sectional study design was chosen as it has been proven to effectively and efficiently sample vaccine acceptance and hesitancy rates at a specific timepoint: 4 months after commencement of staff vaccination exercise and ongoing large-scale rollout of vaccination to the entire Singapore population. As it had been 4 months since the start of staff vaccination, this questionnaire measured actual vaccine acceptance or refusal rather than mere intention. For 4 weeks, between 12th May and 8th June 2021, a structured anonymous self-administered questionnaire was sent via electronic mail to all 1182 HCW, which included doctors, nurses, allied health professionals, operations and administrative personnel. Participation was voluntary. Participants were provided with information on the purpose of research, its voluntary nature, inherent benefits and risks of participation and details on data storage, utility and confidentiality in the informed consent form at the start of the questionnaire. To increase participation rate, 2 additional reminders were sent on the last week of data collection. Informed consent was implied upon voluntary submission of questionnaire.

### Survey questionnaire

We collected demographic characteristics, profession, number of years working in healthcare, residential status, presence of chronic medical conditions, self-perceived risk of acquiring COVID-19 and previous influenza vaccination. HCW were invited to categorise their vaccination status as ‘completed 2 doses’, ‘completed 1 dose’, ‘keen, awaiting vaccination’, or ‘not keen or unable to be vaccinated’. HCW who indicated the affirmative in the first 3 categories were asked to rank their top 5 reasons for taking the vaccination. Survey questions were based on input and discussion of co-authors with expertise in qualitative research, infectious disease and vaccine hesitancy, after cross referencing with available systematic reviews [8–14]. Those who selected ‘not keen or unable to be vaccinated’ were asked to select their reasons for not taking the vaccine, their current sentiments and answer the 5C scale on psychological antecedents of vaccination, a validated 15-item questionnaire which include areas such as confidence, complacency, constraints, calculation and collective responsibility [18]. This framework was selected as the model was easy to implement, reliable and reproducible. The authors believed that this model was the most suitable for HCW as it focused less on knowledge and accessibility to vaccines, superior compared to other scales to explain variance of self-reported vaccine behaviours and tested by a study done among Kuwaiti HCW [19].

For content validation, 5 domain experts on vaccinations within primary care gave feedback to examine the extent to which the question items on the questionnaire were representative of the entire domain the questionnaire seeks to measure. They were excluded from the main study. The questionnaire was revamped and underwent face validation to determine if targeted respondents understood the question items; this is done through recruitment of 10 HCW from different departments to read the informed consent form and perform the questionnaire, and were also excluded from the main study. All excluded HCW were not sent the anonymous questionnaire, therefore these numbers were not included in the total count of HCW (*n* = 1182). Their feedback was used to create the final version of the questionnaire, appended in Annex below.

The guidance for indications and contraindications to COVID-19 vaccine varied throughout the course of the year. According to the prevailing Ministry of Health (MOH) of Singapore guidelines on 31st May 2021 [20], patients with severe cutaneous adverse reactions, active cancer on treatment, allergies and anaphylaxis to non-vaccine triggers, pregnant or breastfeeding were allowed to proceed with COVID-19 vaccination. After the change, all HCW (*n* = 29) who were previously contraindicated and excluded from this study were eligible to be vaccinated.

### Ethical considerations

The study, analysis and publication of results was approved by the NHG Domain Specific Review Board (DSRB). Consent was presumed when participants participated through the act of answering and submitting the questionnaire.

### Statistical analysis

All analyses were conducted using IBM SPSS Statistics Version 22.0. Variables in this analysis included age, sex, ethnicity, profession, years in healthcare, living status (living alone or with others), presence of chronic diseases, perceived risk of acquiring COVID-19 and its exposure, influenza vaccination status, factors associated with vaccine acceptance and hesitancy. HCW were regrouped into the following 4 categories based on their nature of their work: nursing, allied health, administrative and medical. Variables associated with vaccine acceptance and hesitancy were assessed through Pearson’s chi-squared test to provide *p* values. For all statistically significant results, we planned to perform a forced-entry multivariate logistic regression introducing all covariates with a *p* value < 0.05 to identify covariates independently associated with acceptance or hesitancy of the COVID-19 vaccine to provide crude odds ratio (OR) and 95% confidence intervals (C.I.). The top reasons ranked by HCW for vaccine acceptance were also analysed to look for associations, using Pearson’s chi-squared test to provide *p* values and OR if these results were significant. HCW who were not keen or unable to be vaccinated were asked to fill in a 7-point Likert scale for all 15 questions of the 5C scale on the 5 psychological antecedents of vaccination. This was converted to a score (Strongly disagree ➔ 0, Neutral ➔ 4, strongly agree ➔ 7), averaged and tabulated across the 5 areas of the 5C scale. The exceptions were confidence and collective responsibility, for which the scores were reversed. Due to the small group of these HCW, the Likert scale was regrouped into 3 categories: disagree, neutral, agree, for easier analysis.

## Results

### Basic demographics

**Fig. 1:**
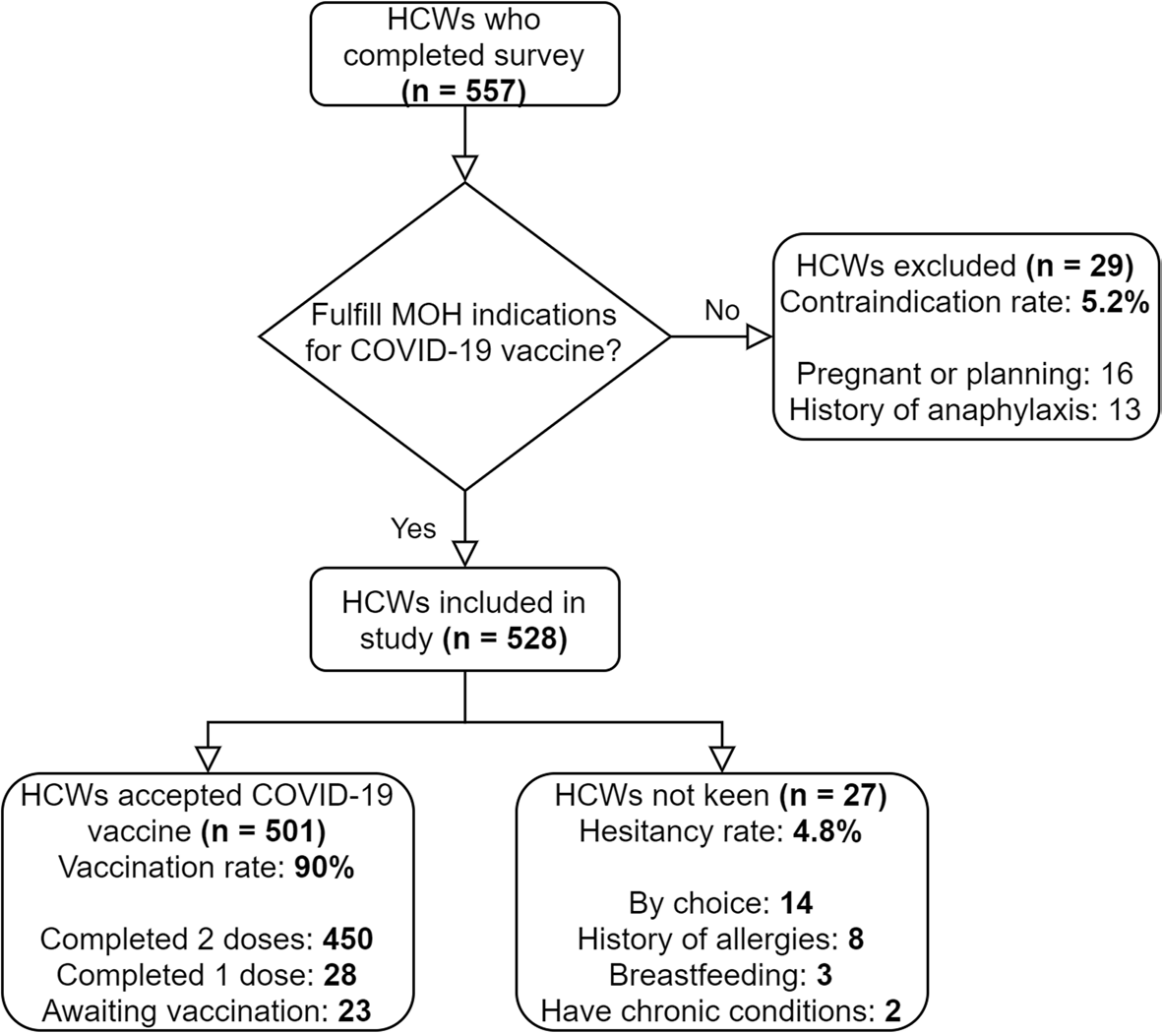
Study inclusion and exclusion

Out of 1182 eligible HCW, 557 respondents completed the survey with a response rate of 47.1%. 29 (5.2%) were excluded due to absolute contraindications to COVID-19 vaccination during that time (pregnant or planning for pregnancy and previous history of anaphylaxis). Of the remaining 528 respondents, 450 (85.2%) were fully vaccinated, 28 (5.3%) had completed 1 dose, 23 (4.4%) were keen and awaiting vaccination, and 27 (5.1%) were not keen to be vaccinated (Fig. 1). Social demographic information can be found in Table 1. Most respondents (*n* = 463, 87.7%) were female and of Chinese ethnicity (*n* = 378, 71.6%). Their occupation spanned the entire spectrum of primary care, including nurses (*n* = 203, 38.4%), allied health professionals (*n* = 142, 26.9%), administrative personnel (*n* = 128, 24.2%) and doctors (*n* = 55, 10.4%). A third (33.3%) of HCW have worked less than 5 years in healthcare, and almost half (47.6%) worked 6–15 years.

**Table 1:** Socio-demographics of respondents on COVID-19 vaccine acceptance

Variables	Respondents (%)	COVID-19 vaccine acceptance		
		Acceptance (%) (*n* = 501)	Hesitancy (%) (*n* = 27)	*P* value
*Sex*				
Female	463 (87.7)	437 (94.4)	26 (5.6)	0.162
Male	65 (12.3)	64 (98.5)	1 (1.5)	
*Age group*				
21–25 years old	50 (9.5)	50 (100)	0 (0)	0.267
26–34 years old	190 (36.0)	178 (93.7)	12 (6.3)	
35–44 years old	138 (26.1)	131 (94.9)	7 (5.1)	
45–54 years old	98 (18.6)	91 (92.9)	7 (7.1)	
≥55 years old	52 (9.8)	51 (98.1)	1 (1.9)	
*Ethnicity*				
Chinese	378 (71.6)	357 (94.4)	21 (5.6)	0.732
Malay	78 (14.8)	75 (96.2)	3 (3.8)	
Indian	43 (8.1)	42 (97.7)	1 (2.3)	
Others	29 (5.5)	27 (93.1)	2 (6.9)	
*Profession*				
Nursing	203 (38.4)	188 (92.6)	15 (7.4)	0.117
Allied Health^a^	142 (26.9)	137 (96.5)	5 (3.5)	
Administrative^b^	128 (24.2)	121 (94.5)	7 (5.5)	
Medical^c^	55 (10.4)	55 (100)	0 (0)	
*Number of years working in healthcare*				
≤5 years	176 (33.3)	166 (94.3)	10 (5.7)	0.439
6–10 years	136 (25.8)	130 (95.6)	6 (4.4)	
11–15 years	115 (21.8)	111 (96.5)	4 (3.5)	
16–20 years	44 (8.3)	39 (88.6)	5 (11.4)	
21–25 years	32 (6.1)	30 (93.8)	2 (6.3)	
26–30 years	13 (2.5)	13 (100)	0 (0)	
≥31 years	12 (2.3)	12 (100)	0 (0)	

^a^Category includes care coordinators, dieticians, financial counsellors, medical social workers, pharmacists, physiotherapists, podiatrists and radiographers

^b^category includes operation executives, call centre operators, patient care and service associates, IT support and temperature screening assistants

^c^Category includes medical officers, residents, family physicians and dentists

From Table 2, 472 (89.4%) of HCW lives with others. 102 (19.3%) HCW have chronic diseases or previous surgeries, of which 10 (9.8%) declared that they were not keen to vaccinate due to their conditions or medications. 424 (80.3%) felt that they were at higher risk of acquiring COVID-19 from their jobs. Majority (*n* = 313, 59.3%) had come into contact with suspected or confirmed COVID-19 patients, and were involved in COVID-19 operations or duties (*n* = 295, 55.9%). Among them, 96.3% (*n* = 284) were confident on the effectiveness of personal protective equipment. In terms of other vaccinations, 92.2% (*n* = 487) had received the influenza vaccination in the past year.

**Table 2:** Factors associated with COVID-19 vaccine acceptance

Variables	Respondents (%)	COVID-19 vaccine acceptance		
		Acceptance (%) (*n* = 501)	Hesitancy (%) (*n* = 27)	*P* value
*Living status*				
Living with others	472 (89.4)	446 (94.5)	26 (5.5)	0.232
Living alone	56 (10.6)	55 (98.2)	1 (1.8)	
*Chronic diseases or previous surgeries*				
Yes	102 (19.3)	95 (93.1)	7 (6.9)	0.372
No	426 (80.7)	406 (95.3)	20 (4.7)	
*Perceived high risk of getting COVID-19 from job*				
Yes	424 (80.3)	406 (95.8)	18 (4.2)	0.067
No	104 (19.7)	95 (91.3)	9 (8.7)	
*Contact with suspected or confirmed COVID-19 patients*				
Yes	313 (59.3)	299 (95.5)	14 (4.5)	0.420
No	215 (40.7)	202 (94.0)	13 (6.0)	
*Involved in clinic, dormitory or isolation facility COVID-19 duties*				
Yes	295 (55.9)	280 (94.9)	15 (5.1)	0.973
No	233 (44.1)	221 (94.8)	12 (5.2)	
*Influenza vaccination in past 1 year*				
Yes	487 (92.2)	462 (94.9)	25 (5.1)	0.943
No	41 (7.8)	39 (95.1)	2 (4.9)	

### Variables associated with COVID-19 vaccine acceptance

There were no significant associations between COVID-19 vaccine acceptance with sex, age, ethnicity, profession or number of years in healthcare (Table 1). There were also no significant associations between COVID-19 vaccine acceptance and living alone, presence of chronic diseases, self-perceived risk of getting COVID-19, contact with suspected or confirmed COVID-19 patients, being involved in COVID-19 operations and duties, perceived effectiveness of personal protective equipment or previous influenza vaccination (Table 2). As there were no statistically significant associations, multivariate logistic regression was not performed.

**Fig. 2:**
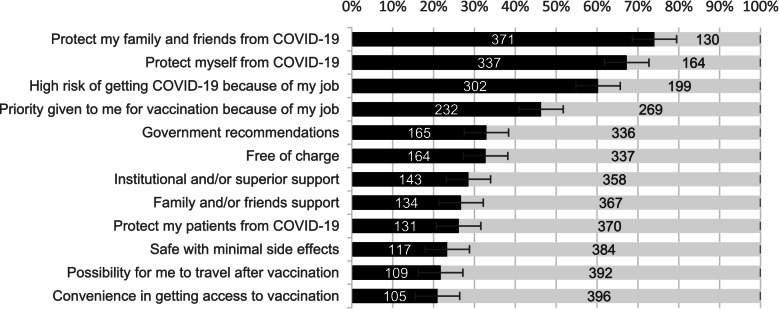
Reasons for COVID-19 vaccine acceptance

According to Fig. 2, the cumulative top 3 reasons for COVID-19 vaccine acceptance ranked by HCW were ‘protect my family and friends from COVID-19’, ‘protect myself from COVID-19’ and ‘high risk of getting COVID-19 because of my job’ (standard deviations as depicted). Each of these 3 reasons were chosen by more than 50% of HCW when asked to rank their top 5 reasons for vaccine acceptance.

Among 501 HCW, living with others was significantly associated with vaccine acceptance to protect themselves from COVID-19 (χ^2^ = 4.54, *p* = 0.033). HCW living with others were 1.8 times more likely to accept vaccine to protect themselves from COVID-19 (OR = 1.84, 95% C.I. 1.04–3.25).

Lack of chronic diseases or previous surgeries was significantly associated with vaccine acceptance to protect themselves from COVID-19 (χ^2^ = 11.4, *p* < 0.001). HCW without chronic diseases or previous surgeries were 2.2 times more likely to take the COVID-19 vaccine to protect themselves (OR = 2.17, 95% C.I. 1.38–3.42).

Contact with COVID-19 patients was significantly associated with vaccine acceptance due to the high risk HCW face on their jobs (χ^2^ = 41.9, *p* < 0.001). Compared to HCW without contact with COVID-19 patients, HCW with suspected or confirmed COVID-19 exposure were 3.4 times more likely to accept vaccination due to the high risk they face at work (OR = 3.38, 95% C.I. 2.32–4.93).

### Factors contributing to COVID-19 vaccine hesitancy

**Fig. 3:**
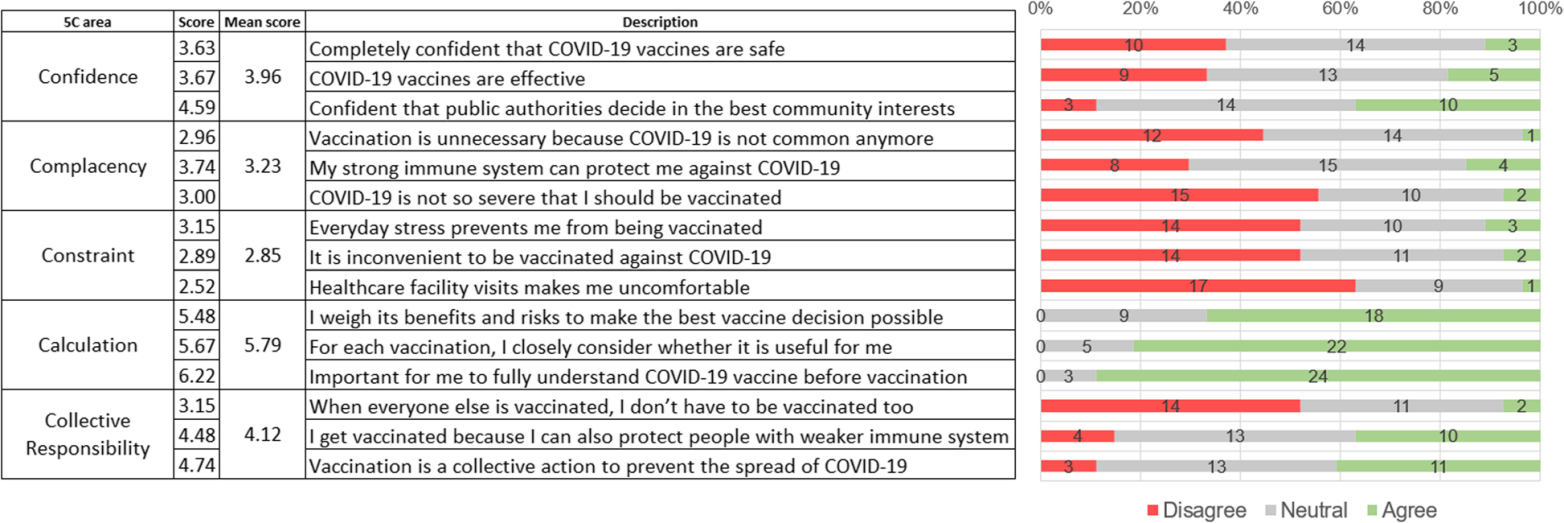
5C psychological antecedents of vaccination (*n* = 27)

The 15-item questionnaire adapted from the 5C psychological antecedents of vaccination was completed by vaccine hesitant HCW (*n* = 27) and illustrated in Fig. 3. The individual and mean scores for the components of the 5Cs, ‘Confidence’ (3.96), ‘Complacency’ (3.23), ‘Constraint’ (2.85), ‘Calculation’ (5.79) and ‘Collective responsibility’ (4.12) were tabulated. Low ‘Complacency’ and ‘Constraint’ scores represented that HCW were not complacent, and does not feel constrained to access to vaccination. Questions pertaining to ‘Calculation’ acquired the highest mean scores with no HCW choosing disagree to any of the 3 statements. This highlighted that HCW would consider the utility, analyse benefits of COVID-19 vaccination and fully understand them before accepting vaccination, in order to make the best decision possible.

## Discussion

Improving COVID-19 vaccination rates can certainly hasten the end of the pandemic, resumption to normal activities and relaxation of vaccine-differentiated safe management measures. To date, few studies have investigated vaccine acceptance specifically among HCW, and most published on intention rather than actual uptake rate. As this was a cross-sectional study conducted in May 2021, vaccine uptake can differ at various timepoints, complicated by changes in vaccine guidance, local infection rates and influenced by legislation on social restrictions. This study managed to mitigate acquiescence bias by usage of a validated 5C psychological antecedents of vaccination questionnaire and thorough content and face validation by HCW from different professions. As it was an anonymous survey, social desirability bias was reduced as participants were free to answer the questionnaire without needing to conform to acceptable norms. Arguably the most significant limitation of our study is sampling and non-response bias. Cross-referencing with internal vaccinations database in September 2021, the primary healthcare cluster has achieved 90% full vaccination rate, which was comparable with 95% as uncovered in this study. However, 52.9% of HCW did not answer the questionnaire; the true hesitancy rate is likely higher than 5.1% as non-respondents are more likely to be hesitant. This is despite the fact that HCW were given ample time of 4 months from start of vaccination campaign with various opportunities and reminders to complete their vaccinations. Overall, the respondents were a good mix of HCW from different professions, gender, races and across different ages. There was an expected significantly greater proportion of females particularly from the nursing profession. While there was a significant proportion of excluded HCW being female (self-selected due to vaccine contraindications at that time i.e. pregnancy, breastfeeding, anaphylaxis), this exclusion (*n* = 29) did not affect the proportion of HCW in terms of gender in the final number of respondents (*n* = 528) for analysis. The profession of HCW sampled was in similar proportions compared to the target population. Given the number of respondents (*n* = 528), this would be representative of public primary care HCW in Singapore.

This study also explored potential reasons for vaccine acceptance among HCW, allowing others to develop key strategies in targeting such factors to improve acceptance. Our study yielded no significant associations between COVID-19 vaccine acceptance and socio-demographic factors, profession, self-perceived risk or presence of chronic diseases. We suspected this was due to well-educated HCW stemming from a developed, multicultural society with strong governmental influence and a possible sampling bias. Moreover, in our sampled population, 95% of HCW were vaccinated (therefore yielding non-significant results due to small numbers of unvaccinated HCW).

The study team wanted to eliminate primacy bias by asking respondents for their top 5 reasons for vaccine acceptance instead of ranking them. HCW quoted most important reasons were to protect themselves, family and friends from COVID-19 and self-perceived high risk due to their jobs (Fig. 2). They prioritised personal reasons over other reasons like altruistic factors (protect patients from COVID-19) or external pressures (governmental, institutional, family or peer support). HCW are not only usually the first to receive vaccinations, they also act as role models to the public, therefore their reasons for vaccine acceptance must be strongly considered as this can help inform future vaccination strategies. Greater contact with community and increased patient encounters may explain higher vaccine intention rates noted among primary care HCW [21].

While the options for factors affecting vaccine acceptance were varied, there were no framework or methods to identify additional factors that may be unique to COVID-19 and this population. Some associations for reasons for vaccine acceptance discovered in our study were similar to other studies performed worldwide. For example, HCW who were exposed to suspected or confirmed COVID-19 cases were 3.4 times more likely to indicate high COVID-19 risk because of their job as one of the top 5 reasons for accepting vaccination. This was noted in an Egyptian study, where risk of COVID-19 was their top reason for vaccine acceptance among HCW [22]. In fact, a scoping review discovered that direct patient contact or higher self-perceived risk were highlighted as associations with greater vaccine acceptance in majority of studies conducted among HCW [11]. Therefore, we can infer that community COVID-19 vaccination rate may increase together with higher disease prevalence. This, in turn, can increase effectiveness of disease prevention through widespread COVID-19 vaccination, and more studies in this area to examine this longitudinal relationship can shed light on the strength of this possible correlation. A Chinese study also noted that vaccination request by employer may improve vaccine acceptance [23]. However, our HCW did not rank government recommendations, institutional or superior support as one of the top 5 reasons for vaccine acceptance, signifying cultural and societal differences which may result in this discrepancy. In summary, factors affecting COVID-19 vaccine acceptance seemed different from other factors discovered in influenza and other vaccine studies, likely because of accelerated vaccine development and unknown adverse effects [8]. A qualitative study may be able to broaden the factors and uncover inherent reasons affecting vaccine hesitancy.

HCW living with others were more likely to rank ‘protecting themselves from COVID-19’ as top reasons for vaccine acceptance, possibly related to high self-perceived risk as mentioned earlier. Interestingly, the absence of chronic diseases was significantly associated with ranking ‘protecting themselves from COVID-19’ as top reasons for vaccine acceptance. Reasons of this association could be confounded by low vaccine confidence among HCW with chronic diseases (unknown adverse reactions, medication interactions), which have been noted in some studies worldwide [24, 25].Vaccine hesitancy may vary over time as additional information about risks and safety become more widely available. ‘Calculation’ recorded the highest mean scores among all the domains under the 5C psychological antecedents of vaccination, as 24 out of 27 wanted fully understand the COVID-19 vaccine before making their decision (Fig. 3). This result was expected as HCW had access to the most knowledge and understanding to the severity and repercussions of the infection. The Kuwaiti study who also used the 5C psychological antecedents revealed that high scores in ‘Confidence’, ‘Constraints’, ‘Calculations’ and ‘Collective Responsibility’ were significantly associated with vaccine acceptance [19]. This scale was also validated to predict COVID-19 vaccine acceptance [14, 26]. We were unable to compare these studies with our study results as we only applied the 5C psychological antecedents to HCW who were hesitant. The sample size for vaccine hesitant group (*n* = 27) is too small for any significant analysis. High mean ‘Calculation’ score of 5.79 (Fig. 3) seemed to be inversely related with vaccine intent, as noted by other studies [27]. However, such studies tend to view vaccine hesitancy as a binary categorical variable instead of a continuum, as discovered in this study on booster hesitancy, which creatively measured this concept as number of days delayed from vaccine eligibility [28]. Future studies can measure the 5C scale at different timepoints to determine the change in psychological sentiments and correlate this with days delayed as a proxy to measure vaccine hesitancy to examine how certain fields (like ‘Calculation’) would be influenced with the change in time. HCW were also the first residents in Singapore prioritised to receive the novel COVID-19 vaccine since mid-January 2021. Based on the theory of Diffusion of Innovation [29], we suspect that the hesitant group may accept vaccination over time, as vaccination campaigns ramped up nationwide. Hesitancy may be sensitive to time-varying infection, government restrictions and mortality rate of the ongoing pandemic. This is also accelerated by nationwide government-led efforts in promoting vaccinations through all media forms.

## Conclusion

In conclusion, it is reassuring that COVID-19 vaccine hesitancy is a minute issue among Singapore primary HCW, having achieved close to 95% acceptance rate with 5% hesitancy rate. This could potentially be contributed by high self-perceived risk and positive contact with COVID-19 patients, as listed by HCW as one of the top reasons for vaccine acceptance. HCW bridge the gap between health care authorities and patients and have a disproportionate influence on patients’ vaccine decisions. Therefore, high vaccination rates correlate positively with their willingness to recommend COVID-19 vaccination to their patients, and strategies that work for HCW can certainly be extrapolated to the community [30]. Further cross-sectional studies can be conducted at different timepoints to evaluate vaccine hesitancy as a continuum instead of a binary categorical variable, and correlate with mean scores derived from the 5C scale. As COVID-19 continues to spread throughout the world, it will be interesting to also study acceptance and hesitancy rates with emergence of the delta and omicron variants and rollout of booster vaccinations.

## Supplementary Information

**Additional file 1.**

**Additional file 2.**

## Data Availability

The datasets generated and/or analysed during the current study are not publicly available due to confidentiality agreement signed by the participants but are available from the corresponding author on reasonable request.

## Abbreviations

COVID-19: Coronavirus Disease 2019
HCW: Healthcare workers
NHG: National Healthcare Group
DSRB: Domain Specific Review Board
WHO: World Health Organisation
MOH: Ministry of Health
