# Supplementary material for: COVID-19 vaccine acceptance and hesitancy among primary healthcare workers in Singapore
Source: BMC Prim Care. 2022 Apr 15;23:81. doi: 10.1186/s12875-022-01693-z (PMC9010198; doi:10.1186/s12875-022-01693-z)
Supplement: Supplementary file 1 — Additional file 1. [file 12875_2022_1693_MOESM1_ESM.pdf]

NUHS
National University
Health System

Survey on COVID-19 Vaccine Hesitancy in NUP

15 mins estimated time to complete

Instructions

INFORMED CONSENT FORM
1. Study Information
Protocol Title: COVID-19 Vaccine Hesitancy among Primary Care Healthcare Workers in Singapore

2. Purpose of the Research Study
You are invited to participate in a research study. It is important to us that you first take time to read through and understand the information provided in this sheet. After you are properly satisfied that you understand this study, and that you wish to take part in the study, you may proceed to complete and submit the questionnaire.
You are invited to participate because we want to understand the factors contributing to COVID-19 vaccine hesitancy among healthcare workers working at National University Polyclinics (NUP). Vaccine hesitancy stands in the way of widespread inoculation efforts which could potentially stop the current epidemic. With this new data, programmes and measures can then be planned to increase vaccine uptake.
This study will involve all healthcare workers who have been offered the COVID-19 vaccine after the vaccination exercise has ended.

3. What procedures will be followed in this study
If you agree to participate in this study, you will be asked to answer a questionnaire. The questionnaire will take about 10 to 15 minutes of your time. The questionnaire is self-administered. This is a dual time-point study and the questionnaire will be administered today. Subjected to the study team's decision and research outcome, the questionnaire may be repeated in 6 to 12 months to re-assess your perceptions and attitudes towards the COVID-19 vaccine.
The questionnaire consists of questions which will explore reasons for taking the vaccination, contraindications to vaccination, self-perceived risk of acquiring COVID-19 and questions based on the 5C scale on the 5 psychological antecedents of vaccination.

4. Your Responsibilities in This Study
If you agree to participate in this study, you may proceed to start filling up the questionnaire.

5. What Is Not Standard Care or is Experimental in This Study
The study is being conducted because we want to understand more about your knowledge and perception of COVID-19 vaccination. The study involves answering a questionnaire. It does not affect the treatment and care which you will be receiving.

6. Possible Risks and Side Effects
This is an observational cross-sectional study involving the administering of a questionnaire. As this study involves the administration of a single questionnaire, physical harm is extremely unlikely. You may feel uncomfortable answering some questions. You may contact the Principal Investigator and be referred to a counsellor or Medical Social Worker should the need arise. Confidentiality of vaccination preferences will be maintained as filling up form via FormSG ensures strict anonymity.

7. Possible Benefits from Participating in the Study
There is no direct benefit from participation in this study. However, your

participation in this study will add to the knowledge of vaccine hesitancy with regards to the COVID-19 vaccine. Your responses would help policy makers to formulate suitable programmes and measures to encourage COVID-19 vaccine uptake amongst Singaporeans, and other healthcare workers.

8. Alternatives to Participation
If you choose not to take part in this study, you will not be discriminated against or identified.

9. Costs & Payments if Participating in the Study
There are no charges involved when you agree to participate in the study.

10. Voluntary Participation
Your participation in this study is voluntary. You may stop participating in this study at any time. Your decision to not take part in this study or to stop your participation will not affect any benefits to which you are entitled. If you decide to stop taking part in this study, you should tell the Principal Investigator or not submit the questionnaire.
However, the data that have been collected until the time of your withdrawal will be kept and analyzed. The reason is to enable a complete and comprehensive evaluation of the study.
In the event of any new information becoming available that may be relevant to your willingness to continue in this study, you (or your legally acceptable representative, if relevant) will be informed in a timely manner by the Principal Investigator or his/her representative.

11. Confidentiality of Study and Medical Records
Your participation in this study will not involve the collection of “Personal Data”. “Personal Data” means data about you which makes you identifiable (i) from such data or (ii) from that data and other information which an organisation has or likely to have access. This includes medical conditions, medications, investigations and treatment history.
Information collected for this study will be kept confidential. Your records, to the extent of the applicable laws and regulations, will not be made publicly available.
However, National University Polyclinics, Domain Specific Review Board and

Ministry of Health will be granted direct access to your information to check study procedures and data, without making any of your information public.
You will not be asked for any identifiable details such as NRIC, Name or Date of Birth. In order to protect your identity and the confidentiality of your information, data will be collected, stored and encrypted by form.gov.sg. Data collected and entered into the organization electronic system are the property of National University Health System. Access to this information will be strictly controlled with the aid of a security code system, restricted to members of the study team. In the event of any publication regarding this study, your identity will remain confidential.
By participating in this research study, you are confirming that you have read, understood and consent to the Personal Data Protection Notification available at https://www.nup.com.sg/Pages/PDPA.aspx (https://www.nup.com.sg/Pages/PDPA.aspx)

12. Who To Contact if You Have Questions
If you have questions about this research study, you may contact the Principal Investigator,
Dr Sky Koh
Bukit Batok Polyclinic
50 Bukit Batok West Avenue 3
Singapore 659164
sky_wc_koh@nuhs.edu.sg (mailto:sky_wc_koh@nuhs.edu.sg)

The study has been reviewed by the NHG Domain Specific Review Board (the central ethics committee) for ethics approval.
If you want an independent opinion to discuss problems and questions, obtain information and offer inputs on your rights as a research subject, you may contact the NHG Domain Specific Review Board Secretariat at 6471-3266. You can also find more information about participating in clinical research, the NHG Domain Specific Review Board and its review processes at www.research.nhg.com.sg (http://www.research.nhg.com.sg).
If you have any complaints or feedback about this research study, you may contact the Principal Investigator or the NHG Domain Specific Review Board Secretariat.
If you require further information on our Data Protection Policy or clarification

If you require further information on our Data Protection Policy or clarification related to data protection, please contact us at:
Data Protection Office
Email: NUP_DPO@nuhs.edu.sg (mailto:NUP_DPO@nuhs.edu.sg)

I voluntarily consent to take part in this research study. I have fully discussed and understood the purpose and procedures of this study. This study has been explained to me in a language that I understand. I have been given enough time to ask any questions that I have about the study, and all my questions have been answered to my satisfaction. I have also been informed and understood the alternative treatments or procedures available and their possible benefits and risks.
By participating in this research study, I confirm that I have read, understood and consent to the National University Polyclinics Personal Data Protection Notification.

---

version 1.5 updated 20/04/21

1. **What is your COVID-19 vaccination status?**

Completed 2 doses, fully vaccinated ▾ ✕

Demographics

2. **Gender**

- ○ Male
- ○ Female

3. **Age**

- ○ 21-25

- ○ 26-34
- ○ 35-44
- ○ 45-54
- ○ 55-64
- ○ >65

4. Race

- ○ Chinese
- ○ Malay
- ○ Indian
- ○ Others

If others, please specify.

5. Department

- ○ Medical
- ○ Nursing
- ○ Operations (includes Temperature Screening Assistant, Patient Care Assistant, Patient Service Associate, Ops Executives & Managers)
- ○ Allied Health (non-nursing care coordinators, Dietician, FC, MSW, Podiatry, Physio, Psychologist)
- ○ Diagnostics (NHGD)
- ○ Pharmacy (NHGPh)

- ○ HQ Staff
- ○ IT Support
- ○ ISS (housekeeping)
- ○ Misc (F&B providers and vendors etc)
- ○ Others

If others, please specify.

6. No of years working in healthcare (or polyclinic)

- ○ ≤5 years
- ○ 6-10
- ○ 11-15
- ○ 16-20
- ○ 21-25
- ○ 26-30
- ○ ≥ 31 years

7. Residential status: Are you living alone?

If you are living with someone else, please input in the next question

✕ NO ✓ YES

8. I am living with

Select all that apply.

- ☐ My spouse/partner

- [ ] My child/children
- [ ] My grandparent (s)
- [ ] My parent (s)
- [ ] My grandchild/grandchildren
- [ ] My housemates who are healthcare workers
- [ ] My housemates who are non-healthcare workers

9. I have chronic disease (s) or have had previous surgery (s)

✕ NO	✓ YES

10. Because of my condition or my medications, I am not keen to receive the COVID-19 vaccine.

✕ NO	✓ YES

Factors associated with COVID-19 vaccine acceptance and hesitancy

11. I have a high risk of contracting COVID-19 because of my job.

✕ NO	✓ YES

12. Because of my job, I have come into contact with suspected or confirmed COVID-19 patients.

✕ NO	✓ YES

13. I am/was involved in red zone/flu cluster duties at the Polyclinic or volunteered at Dormitory/Swab Isolation Facility during the COVID-19 pandemic.

✕ NO	✓ YES (selected)

14. The Personal Protective Equipment (PPE) at my workplace was effective in protecting me from contracting COVID-19.

✕ NO	✓ YES

15. I had received influenza vaccine in the past year.

✕ NO	✓ YES

16. What are your top 5 reasons for opting to receive the COVID-19 vaccine?

- ☐ Family and/or friends support
- ☐ Institutional and/or superior support
- ☐ Government recommendations
- ☐ Free of charge
- ☐ Safe with minimal side effects
- ☐ High risk of getting COVID-19 because of my job
- ☐ Priority given to me for vaccination because of my job
- ☐ Convenience in getting access to vaccination
- ☐ Protect myself from COVID-19
- ☐ Protect my family and friends from COVID-19

- [ ] Protect my patients from COVID-19
- [ ] Of vulnerable age group or have chronic diseases
- [ ] Inability to get it in my home country as easily (for non-residents)
- [ ] Ability to take time off work (or MC) for vaccination
- [ ] Possibility for me to travel after vaccination
- [ ] Approval by religion
- [ ] Others

If others, please specify.

SUBMIT

POWERED BY

Privacy Policy Terms of Use

protected by reCAPTCHA

Privacy - Terms
