# Supplementary material for: COVID-19 vaccine acceptance and hesitancy among primary healthcare workers in Singapore
Source: BMC Prim Care. 2022 Apr 15;23:81. doi: 10.1186/s12875-022-01693-z (PMC9010198; doi:10.1186/s12875-022-01693-z)
Supplement: Supplementary file 2 — Additional file 2. [file 12875_2022_1693_MOESM2_ESM.pdf]

NUHS
National University
Health System

# Survey on COVID-19 Vaccine Hesitancy in NUP

**15 mins** estimated time to complete

## Instructions

INFORMED CONSENT FORM

1. Study Information

Protocol Title: COVID-19 Vaccine Hesitancy among Primary Care Healthcare Workers in Singapore

2. Purpose of the Research Study

You are invited to participate in a research study. It is important to us that you first take time to read through and understand the information provided in this sheet. After you are properly satisfied that you understand this study, and that you wish to take part in the study, you may proceed to complete and submit the questionnaire.

You are invited to participate because we want to understand the factors contributing to COVID-19 vaccine hesitancy among healthcare workers working at National University Polyclinics (NUP). Vaccine hesitancy stands in the way of widespread inoculation efforts which could potentially stop the current epidemic. With this new data, programmes and measures can then be planned to increase vaccine uptake.

This study will involve all healthcare workers who have been offered the COVID-19 vaccine after the vaccination exercise has ended.

3. What procedures will be followed in this study
If you agree to participate in this study, you will be asked to answer a questionnaire. The questionnaire will take about 10 to 15 minutes of your time. The questionnaire is self-administered. This is a dual time-point study and the questionnaire will be administered today. Subjected to the study team's decision and research outcome, the questionnaire may be repeated in 6 to 12 months to re-assess your perceptions and attitudes towards the COVID-19 vaccine.
The questionnaire consists of questions which will explore reasons for taking the vaccination, contraindications to vaccination, self-perceived risk of acquiring COVID-19 and questions based on the 5C scale on the 5 psychological antecedents of vaccination.

4. Your Responsibilities in This Study
If you agree to participate in this study, you may proceed to start filling up the questionnaire.

5. What Is Not Standard Care or is Experimental in This Study
The study is being conducted because we want to understand more about your knowledge and perception of COVID-19 vaccination. The study involves answering a questionnaire. It does not affect the treatment and care which you will be receiving.

6. Possible Risks and Side Effects
This is an observational cross-sectional study involving the administering of a questionnaire. As this study involves the administration of a single questionnaire, physical harm is extremely unlikely. You may feel uncomfortable answering some questions. You may contact the Principal Investigator and be referred to a counsellor or Medical Social Worker should the need arise. Confidentiality of vaccination preferences will be maintained as filling up form via FormSG ensures strict anonymity.

7. Possible Benefits from Participating in the Study
There is no direct benefit from participation in this study. However, your

participation in this study will add to the knowledge of vaccine hesitancy with regards to the COVID-19 vaccine. Your responses would help policy makers to formulate suitable programmes and measures to encourage COVID-19 vaccine uptake amongst Singaporeans, and other healthcare workers.

8. Alternatives to Participation
If you choose not to take part in this study, you will not be discriminated against or identified.

9. Costs & Payments if Participating in the Study
There are no charges involved when you agree to participate in the study.

10. Voluntary Participation
Your participation in this study is voluntary. You may stop participating in this study at any time. Your decision to not take part in this study or to stop your participation will not affect any benefits to which you are entitled. If you decide to stop taking part in this study, you should tell the Principal Investigator or not submit the questionnaire.
However, the data that have been collected until the time of your withdrawal will be kept and analyzed. The reason is to enable a complete and comprehensive evaluation of the study.
In the event of any new information becoming available that may be relevant to your willingness to continue in this study, you (or your legally acceptable representative, if relevant) will be informed in a timely manner by the Principal Investigator or his/her representative.

11. Confidentiality of Study and Medical Records
Your participation in this study will not involve the collection of “Personal Data”. “Personal Data” means data about you which makes you identifiable (i) from such data or (ii) from that data and other information which an organisation has or likely to have access. This includes medical conditions, medications, investigations and treatment history.
Information collected for this study will be kept confidential. Your records, to the extent of the applicable laws and regulations, will not be made publicly available.
However, National University Polyclinics, Domain Specific Review Board and

Ministry of Health will be granted direct access to your information to check study procedures and data, without making any of your information public.
You will not be asked for any identifiable details such as NRIC, Name or Date of Birth. In order to protect your identity and the confidentiality of your information, data will be collected, stored and encrypted by form.gov.sg. Data collected and entered into the organization electronic system are the property of National University Health System. Access to this information will be strictly controlled with the aid of a security code system, restricted to members of the study team. In the event of any publication regarding this study, your identity will remain confidential.
By participating in this research study, you are confirming that you have read, understood and consent to the Personal Data Protection Notification available at https://www.nup.com.sg/Pages/PDPA.aspx (https://www.nup.com.sg/Pages/PDPA.aspx)

12. Who To Contact if You Have Questions
If you have questions about this research study, you may contact the Principal Investigator,
Dr Sky Koh
Bukit Batok Polyclinic
50 Bukit Batok West Avenue 3
Singapore 659164
sky_wc_koh@nuhs.edu.sg (mailto:sky_wc_koh@nuhs.edu.sg)

The study has been reviewed by the NHG Domain Specific Review Board (the central ethics committee) for ethics approval.
If you want an independent opinion to discuss problems and questions, obtain information and offer inputs on your rights as a research subject, you may contact the NHG Domain Specific Review Board Secretariat at 6471-3266. You can also find more information about participating in clinical research, the NHG Domain Specific Review Board and its review processes at www.research.nhg.com.sg (http://www.research.nhg.com.sg).
If you have any complaints or feedback about this research study, you may contact the Principal Investigator or the NHG Domain Specific Review Board Secretariat.
If you require further information on our Data Protection Policy or clarification [illegible]

If you require further information on our Data Protection Policy or clarification related to data protection, please contact us at:
Data Protection Office
Email: NUP_DPO@nuhs.edu.sg (mailto:NUP_DPO@nuhs.edu.sg)

I voluntarily consent to take part in this research study. I have fully discussed and understood the purpose and procedures of this study. This study has been explained to me in a language that I understand. I have been given enough time to ask any questions that I have about the study, and all my questions have been answered to my satisfaction. I have also been informed and understood the alternative treatments or procedures available and their possible benefits and risks.
By participating in this research study, I confirm that I have read, understood and consent to the National University Polyclinics Personal Data Protection Notification.

---

version 1.5 updated 20/04/21

1. **What is your COVID-19 vaccination status?**

Not keen or unable to be vaccinated ▾ ✕

2. **What is your reason for not taking up the Covid-19 vaccination?**

Anaphylaxis is a life-threatening allergic reaction with two or more of the following three criteria:
a) Hives or face/eyelid/lip/throat swelling;
b) Difficulty breathing;
c) Dizziness.

- [ ] I do not want to be vaccinated (by choice)
- [ ] Currently pregnant
- [ ] Planning for pregnancy (not keen to defer plans just to receive vaccine)

- [ ] Currently breastfeeding (with no intentions of suspending just to receive vaccine)
- [ ] History of allergy to COVID-19 vaccine components
- [ ] History of anaphylaxis (2 of the following: lip or eye swelling, breathlessness or rashes) to any medications, vaccines, food, insect stings, or unknown trigger
- [ ] History of having been prescribed an Epi-Pen
- [ ] History of single allergy without anaphylaxis
- [ ] History of multiple allergies without anaphylaxis
- [ ] Severe skin conditions - Stevens-Johnsons Syndrome (SJS), Toxic Epidermal Necrolysis (TEN), Drug Rash with Eosinophilia and Systemic Symptoms (DRESS)
- [ ] Active cancer or on cancer treatment
- [ ] On immune therapy
- [ ] Recent transplant in last 3 months
- [ ] Low platelets <50k
- [ ] HIV with CD4 count <200
- [ ] Others

If others, please specify.

## Demographics

3. Gender

- ( ) Male
- ( ) Female

4. Age

- 21-25
- 26-34
- 35-44
- 45-54
- 55-64
- >65

5. Race

- Chinese
- Malay
- Indian
- Others

If others, please specify.

6. Department

- Medical
- Nursing
- Operations (includes Temperature Screening Assistant, Patient Care Assistant, Patient Service Associate, Ops Executives & Managers)
- Allied Health (non-nursing care coordinators, Dietician, FC, MSW, Podiatry, Physio, Psychologist)

- ◯ Diagnostics (NHGD)
- ◯ Pharmacy (NHGPh)
- ◯ HQ Staff
- ◯ IT Support
- ◯ ISS (housekeeping)
- ◯ Misc (F&B providers and vendors etc)
- ◯ Others

If others, please specify.

7. No of years working in healthcare (or polyclinic)

- ◯ ≤5 years
- ◯ 6-10
- ◯ 11-15
- ◯ 16-20
- ◯ 21-25
- ◯ 26-30
- ◯ ≥ 31 years

8. Residential status: Are you living alone?

If you are living with someone else, please input in the next question

| ✕ NO | ✓ YES |
| --- | --- |

9. I am living with

Select all that apply.

- [ ] My spouse/partner
- [ ] My child/children
- [ ] My grandparent (s)
- [ ] My parent (s)
- [ ] My grandchild/grandchildren
- [ ] My housemates who are healthcare workers
- [ ] My housemates who are non-healthcare workers

10. I have chronic disease (s) or have had previous surgery (s)

| ✕ NO | ✓ YES |
|---|---|

11. Because of my condition or my medications, I am not keen to receive the COVID-19 vaccine.

| ✕ NO | ✓ YES |
|---|---|

## Factors associated with COVID-19 vaccine acceptance and hesitancy

12. I have a high risk of contracting COVID-19 because of my job.

| ✕ NO | ✓ YES |
|---|---|

13. Because of my job, I have come into contact with suspected or confirmed COVID-19 patients.

| ✕ NO | ✓ YES |
|---|---|

14. I am/was involved in red zone/flu cluster duties at the Polyclinic or volunteered at Dormitory/Swab Isolation Facility during the COVID-19 pandemic.

| ✕ NO | ✓ YES |
|---|---|

15. The Personal Protective Equipment (PPE) at my workplace was effective in protecting me from contracting COVID-19.

| ✕ NO | ✓ YES |
|---|---|

16. I had received influenza vaccine in the past year.

| ✕ NO | ✓ YES |
|---|---|

## 5C psychological antecedents of vaccination

17. I am completely confident that the COVID-19 vaccines are safe.

- ○ Strongly disagree
- ○ Moderately disagree
- ○ Slightly disagree
- ○ Neutral
- ○ Slightly agree
- ○ Moderately agree
- ○ Strongly agree

18. COVID-19 vaccines are effective.

- Strongly disagree
- Moderately disagree
- Slightly disagree
- Neutral
- Slightly agree
- Moderately agree
- Strongly agree

19. Regarding COVID-19 vaccines, I am confident that our public authorities decide in the best interest of the community.

- Strongly disagree
- Moderately disagree
- Slightly disagree
- Neutral
- Slightly agree
- Moderately agree
- Strongly agree

20. COVID-19 vaccination is unnecessary because COVID-19 is not common anymore.

- Strongly disagree

- Moderately disagree
- Slightly disagree
- Neutral
- Slightly agree
- Moderately agree
- Strongly agree

21. My immune system is strong; it can protect me against COVID-19.

- Strongly disagree
- Moderately disagree
- Slightly disagree
- Neutral
- Slightly agree
- Moderately agree
- Strongly agree

22. COVID-19 is not so severe that I should be vaccinated.

- Strongly disagree
- Moderately disagree
- Slightly disagree
- Neutral
- Slightly agree
- Moderately agree

- Strongly agree

23. Everyday stress prevents me from being vaccinated against COVID-19.

- Strongly disagree
- Moderately disagree
- Slightly disagree
- Neutral
- Slightly agree
- Moderately agree
- Strongly agree

24. For me, it is inconvenient to be vaccinated against COVID-19.

- Strongly disagree
- Moderately disagree
- Slightly disagree
- Neutral
- Slightly agree
- Moderately agree
- Strongly agree

25. Visiting a healthcare facility makes me feel uncomfortable; this keeps me from being vaccinated against COVID-19.

- Strongly disagree

- Moderately disagree
- Slightly disagree
- Neutral
- Slightly agree
- Moderately agree
- Strongly agree

26. When I think about being vaccinated against COVID-19, I weigh its benefits and risks to make the best decision possible.

- Strongly disagree
- Moderately disagree
- Slightly disagree
- Neutral
- Slightly agree
- Moderately agree
- Strongly agree

27. For each and every vaccination, I closely consider whether it is useful for me.

- Strongly disagree
- Moderately disagree
- Slightly disagree
- Neutral

- Slightly agree
- Moderately agree
- Strongly agree

28. **It is important for me to fully understand the COVID-19 vaccine before I get vaccinated.**

- Strongly disagree
- Moderately disagree
- Slightly disagree
- Neutral
- Slightly agree
- Moderately agree
- Strongly agree

29. **When everyone else is vaccinated against COVID-19, I don’t have to be vaccinated too.**

- Strongly disagree
- Moderately disagree
- Slightly disagree
- Neutral
- Slightly agree
- Moderately agree
- Strongly agree

30. **I get vaccinated against COVID-19 because I can also protect people with a weaker immune system.**

- Strongly disagree
- Moderately disagree
- Slightly disagree
- Neutral
- Slightly agree
- Moderately agree
- Strongly agree

31. **Vaccination is a collective action to prevent the spread of COVID-19.**

- Strongly disagree
- Moderately disagree
- Slightly disagree
- Neutral
- Slightly agree
- Moderately agree
- Strongly agree

32. **Choose the statement that best describes your current sentiment.**

- I am currently breastfeeding or planning for pregnancy; it is best to avoid COVID-19 vaccine for now

- ○ I am worried that the COVID-19 vaccine may affect my chronic diseases or interact with the medications I am taking
- ○ I do not trust the safety of existing vaccines that have been approved for distribution because of the lack of long-term safety data available and its speed of development
- ○ I am worried about the side effects and serious adverse events that may occur after vaccination
- ○ I believe the current vaccines may not be effective in the long-term, given the present lack of understanding on the period of effectiveness and/or the efficacy against new variants of the coronavirus
- ○ I will choose to wait for a better vaccine/brand (eg non mRNA vaccine)
- ○ I do not see the urgency to be vaccinated at this stage, given Singapore's management of the Covid-19 situation
- ○ Others

If others, please specify.

SUBMIT

POWERED BY

Privacy Policy Terms of Use

protected by **reCAPTCHA**

Privacy - Terms
